# Transcriptomic and network analysis identifies shared and unique pathways and immune changes across fibrotic interstitial lung diseases

**DOI:** 10.18632/aging.205530

**Published:** 2024-02-12

**Authors:** Wenhao Liu, Kangping Huang, Xin-Zhuang Yang, Ping Wang

**Affiliations:** 1 Eight-Year Program of Clinical Medicine, Peking Union Medical College Hospital, Chinese Academy of Medical Sciences and Peking Union Medical College, Beijing 100730, China; 2 Center for Bioinformatics, National Infrastructures for Translational Medicine, Institute of Clinical Medicine and Peking Union Medical College Hospital, Chinese Academy of Medical Sciences and Peking Union Medical College, Beijing 100730, China; 3 Department of Pulmonary and Critical Care Medicine, Peking Union Medical College Hospital, Chinese Academy of Medical Science and Peking Union Medical College, Beijing 100730, China

**Keywords:** fibrotic interstitial lung diseases, idiopathic pulmonary fibrosis, fibrotic hypersensitivity pneumonitis, connective tissue disease-associated interstitial lung diseases, transcriptome data

## Abstract

Background: Interstitial lung disease (ILD) encompasses a diverse group of disorders characterized by chronic inflammation and fibrosis of the pulmonary interstitium. Three ILDs, namely idiopathic pulmonary fibrosis (IPF), fibrotic hypersensitivity pneumonitis (fHP), and connective tissue disease-associated ILD (CTD-ILD), exhibit similar progressive fibrosis phenotypes, yet possess distinct etiologies, encouraging us to explore their different underlying mechanisms.

Methods: Transcriptome data of fibrotic lung tissues from patients with IPF, fHP, and CTD-ILD were subjected to functional annotation, network, and pathway analyses. Additionally, we employed the xCell deconvolution algorithm to predict immune cell infiltration in patients with fibrotic ILDs and healthy controls.

Results: We identified a shared progressive fibrosis-related module in these diseases which was related to extracellular matrix (ECM) degradation and production and potentially regulated by the p53 family transcription factors. In IPF, neuron-related processes emerged as a critical specific mechanism in functional enrichment. In fHP, we observed that B cell signaling and immunoglobulin A (IgA) production may act as predominant processes, which was further verified by B cell infiltration and the central role of CD19 gene. In CTD-ILD, active chemokine processes were enriched, and active dendritic cells (aDCs) were predicted to infiltrate the lung tissues.

Conclusions: This study revealed shared and specific molecular and cellular pathways among IPF, fHP, and CTD-ILD, providing a basis for understanding their pathogenesis and identifying potential therapeutic targets.

## INTRODUCTION

Interstitial lung disease (ILD) refers to a group of lung diseases characterized by fibrosis and/or inflammation of the lung interstitium. ILD can be idiopathic or associated with known causes such as connective tissue disease (CTD) and environmental exposures. Idiopathic pulmonary fibrosis (IPF), fibrotic hypersensitivity pneumonitis (fHP), and CTD-associated ILD (CTD-ILD) are the most common three subtypes of fibrotic ILD, leading to progressive pulmonary fibrosis and respiratory failure [1]. Therefore, a profound understanding and comparison of their pathogenesis is indispensable.

The pathogenesis of IPF often includes pathological fibrosis in response to chronic lung tissue injury [2]. Fibroblasts and myofibroblasts play the most important roles in IPF, which overproduce extracellular matrix (ECM) through several dysfunctional signaling pathways [3]. Previous studies identified well-established mediators of IPF including transforming growth factor-β (TGF-β), fibroblast growth factor (FGF), CXCL12, CCL2, and tumor necrosis factor-α (TNF-α) [4]. The poor prognosis of IPF necessitates more investigation into its pathogenesis, including interactions of previously known mechanisms and new mechanisms [5].

fHP is a type of ILDs caused by continuous exposure to external antigens. fHP and IPF have some similar clinical, radiological, and pathological features, including progressing fibrosis. As antigens contribute to triggering of fHP, T lymphocytes and granuloma are involved in the pathophysiology of fHP [6], Th1 inflammatory response is canonical in the development of fHP while the Th17 response might be also involved in its pathogenesis [7]. Recent bioinformatic studies found that fHP and IPF have common molecular patterns, and HP-specific genes may be *BGN* and *CXCL9*, which are related to inflammation initiation and granulomatous diseases [8] However, the specific molecular features of fHP remain to be explored.

CTD-ILD is a heterogenous conditions and can exhibit similar clinical and imaging patterns with IPF. Systemic sclerosis, rheumatoid arthritis, and mixed connective tissue disease are the most common conditions that may concur with progressive pulmonary fibrosis. However, the treatment of CTD-ILD and IPF is different, and our understanding of this disparity is limited [9]. Previous mechanistic studies demonstrated that myofibroblasts possess profibrotic and anti-apoptotic properties in systemic sclerosis associated ILD (SSc-ILD), while alveolar epithelial cells play a more significant role in IPF [10]. Immune mechanisms including innate and adaptive immunity, especially profibrotic and anti-fibrotic immune cells, were also reported to play a role in CTD-ILD [11, 12]. So far, no definitive pattern has been identified to distinguish CTD-ILD from IPF, thus it is crucial to evaluate the distinct features of CTD-ILD.

Bioinformatics has provided new insight into different fibrotic ILDs [13–15]. However, many transcriptome studies of lung tissues were limited by small sample sizes and a narrow focus on specific types of fibrotic ILDs, potentially introducing bias. Hence, there is an urgent need for a comprehensive investigation encompassing multiple ILDs with distinct etiologies yet sharing similar pathological patterns. Furthermore, previous studies have not adequately integrated the expression patterns, regulatory mechanisms, and cellular alterations within lung tissue samples. In this study, we utilized various databases to perform network and pathway analyses. We also incorporated transcription factors (TFs) data mining to investigate gene expression regulation patterns, and immune infiltration analysis to discover cellular changes. Through this comprehensive approach, our objective is to elucidate the differences and commonalities of the pathogenesis of the three prevalent fibrotic ILDs, thereby enabling more precise and targeted treatments for patients.

## MATERIALS AND METHODS

### Datasets employed in this study

The microarray datasets used in this study were obtained from the GEO database (http://www.ncbi.nlm.nih.gov/geo/). The criteria for retrieval were: A) samples were from human lung tissue, B) gene expression pattern was profiled, C) datasets contained both patients and healthy individuals without a history of pulmonary fibrosis, D) the diagnosis of IPF was based on standard American Thoracic Society criteria [16], E) The diagnosis of fHP was made according to accepted guidelines or by multidisciplinary consensus using ATS criteria [8, 17], F) The RA-UIP subjects had evidence of UIP on lung biopsy and rheumatoid arthritis (RA) defined by the American College of Rheumatology and European League Against Rheumatism guidelines [18]. In addition, all patients with systemic sclerosis (SSc) met the American College of Rheumatology criteria for the diagnosis of SSc [19].

To ensure the consistency and completeness of the datasets, we manually identified relevant literature using keywords filters and applied R programming language (version: 4.1.3) for subsequent analysis. Finally, IPF dataset (GSE175457) [20], fHP dataset (GSE150910) [8], and CTD-ILD datasets (SSc-ILD: GSE48149 [21, 22]; RA-ILD: GSE199152 [23]) were included, each contributing unique insights into various lung conditions. We merged CTD-ILD datasets and corrected the batch effects using the “combat” function in the SVA package (version: 3.38.0) [24]. Next, we normalized the merged datasets and adjusted for covariates using the “Normalize between arrays” and “remove Batch Effect” functions in the limma package (version: 3.46.0) [25]. Table 1 summarizes the included datasets.

**Table 1:** The information of all the datasets employed in this study.

**Data sets** ^*^	**Data**		**Tissue sources**	**References**	**Category**	**GPL**
	**Case**	**Control**				
GSE48149	44^a^	9	Lung tissue	Hsu et al., 2011; Renaud et al., 2020	CTD-ILD (SSc-ILD)	GPL16221
GSE199152	23^b^	4	Lung tissue	Vassallo et al., 2022	CTD-ILD (RA-ILD)	GPL16791
GSE150910	185^c^	103	Lung tissue	Furusawa et al. 2020	CHP	GPL24676
GSE175457	234	188	Lung tissue	Borie et al., 2022	IPF	GPL24676

^*^: All data sets used in this study contain a total of 646 samples, among which there were 342 cases and 304 controls. All samples were collected in the lung tissue. **^a^**: 23 lung samples from SSc-ILD patients were employed in this study. **^b^**: 3 lung samples from RA-ILD patients were employed in this study. **^c^**: 82 lung samples from CHP patients were employed in this study.

### Identification of differentially expressed genes and functional annotation

To identify differentially expressed genes (DEGs) in lung tissue samples from ILD patients and healthy controls, we performed differential expression analysis using the limma package (version: 3.46.0), controlling for age. The threshold for screening DEGs was |log2 FC (fold change)| > 1 and false discovery rate (FDR) < 0.01. Upregulated and downregulated common DEGs for three ILDs were then subjected to functional annotation.

Enrichment analysis of Gene Ontology (GO) and Disease Ontology (DO) was performed on common DEGs using the clusterProfiler package (version: 3.18.1) [26]. Kyoto Encyclopedia of Genes and Genomes (KEGG) (http://www.genome.jp/kegg/) and gene set enrichment analysis (GSEA) were performed for common DEGs. The threshold for significant differences in the aforementioned enrichment analyses was set at FDR < 0.05.

### Network and pathway analysis

To explore the functional interaction between the common DEGs of IPF, fHP, and CTD-ILD, PPI network analysis was done using the STRINGdb package (version: 2.6.5) with a confidence score of ≥ 0.7 [0,1]. Entrez gene identifiers of DEGs were imported into STRING (Search Tool for the Retrieval of Interacting Genes/Proteins) database (Version: 11.5) [27] for network and pathway analyses. We imported the information of the PPI network into the igraph package (version: 1.12.1) and selected the minimum connected network for further pathway analyses. To explore the unique biological pathways, we individually used the pathway analysis data derived from the KEGG database on the PPI network of IPF, fHP, and CTD-ILD.

### Transcription factors analysis

The common DEGs were imported into Cytospace for network analysis of TFs [28]. RcisTarget package [29] was used to acquire information on TFs and gene targets, and an adjusted P-value < 0.05 was considered significant. Subsequently, we separately identified the unique TFs in IPF, fHP, and CTD-ILD.

### Immune infiltration analysis

The xCell deconvolution algorithm was applied in this study [30]. We used the “immunedeconv” package to comprehensively analyze tissue immune infiltration based on gene expression profiles [31]. Following the application of the xCell deconvolution algorithm, we obtained the estimated proportions of various immune cells in the lung tissues of ILD patients and healthy controls. Student′s t-test was used to investigate the differences in immune cell infiltration between these groups.

### Statistical analysis

For this study, statistical computations were performed using R (version 4.2.2). Microarray data, sourced from the GEO database, were selected based on strict criteria ensuring sample relevance and quality. Differential expression analysis was executed via the limma package, with age as a covariate, identifying DEGs at a |log2 FC| > 1 and FDR < 0.01 threshold. Functional annotations were derived using the clusterProfiler package, with an FDR < 0.05 denoting significance. PPI networks, constructed using STRINGdb with a confidence score ≥ 0.7, were analyzed in igraph to discern minimum connected networks for pathway analysis against KEGG database entries. Transcription factor associations were determined using the RcisTarget package, with significance set at an adjusted P-value < 0.05. Immune cell proportions in lung tissue were estimated using the xCell algorithm within the immunedeconv package, with Student’s t-tests assessing differences in infiltration between patient and control groups. These R scripts for replication of this research can be found in our dedicated online repository at https://github.com/Robinwhliu/IPF-fHP-CTD_ILD.

### Data available statement

Lists of genes with varying expression across three diseases are presented in Supplementary Tables 1–3. Comprehensive gene differential and functional enrichment analyses are included in Supplementary Tables 4–10. The full expression matrix employed in this research can be found in the GEO database (https://www.ncbi.nlm.nih.gov/geo/). The R programming script for reproducing our analyses and graphical representations is also open for download on GitHub at the provided URL (https://github.com/uuihbgg1/IPF-fHD-CTD).

## RESULTS

### Differentially expressed genes

**Figure 1:**
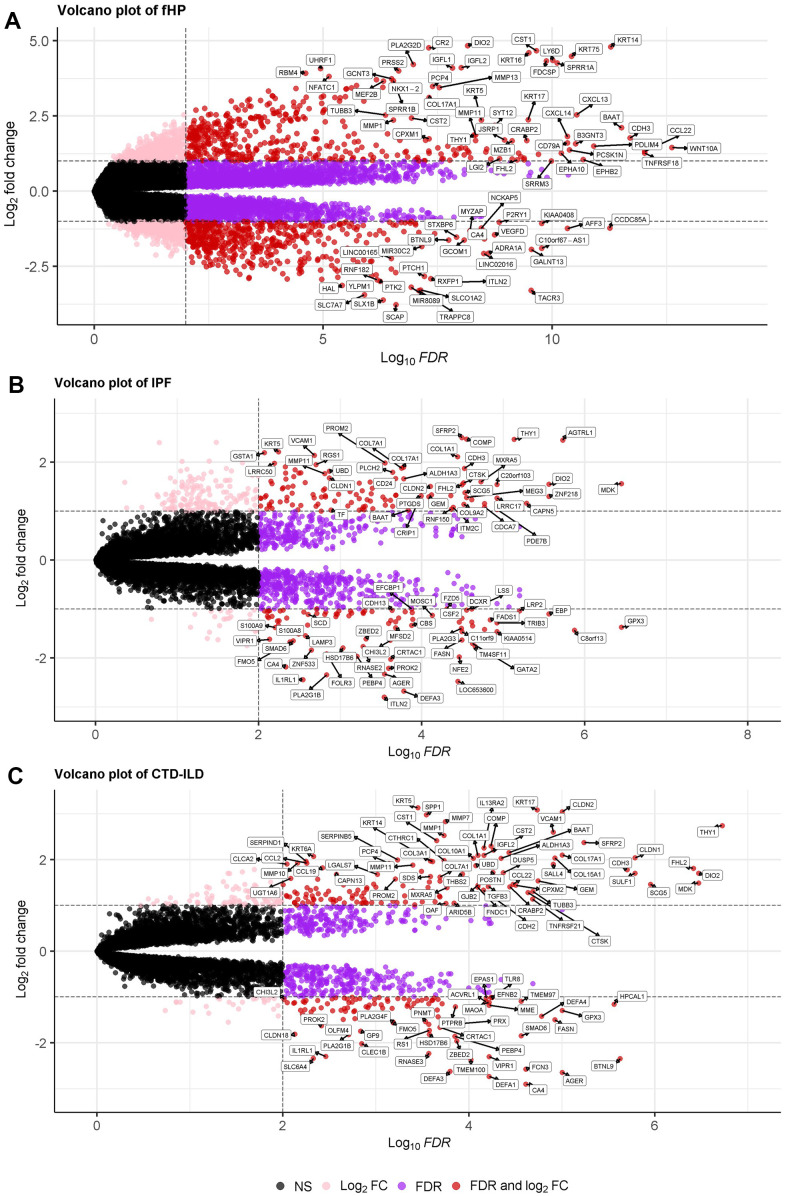
(**A**–**C**) Volcano plot demonstrating an overview of the differential expression of all genes in fHP, IPF and CTD-ILD. The threshold in the volcano plot was -log10 adjusted P>2 and |log2 fold change| >0.5; red dots indicate significant differential expressed genes. Note: CTD-ILD expression matrix was the mergence of SSc-ILD and RA-ILD datasets.

**Figure 2:**
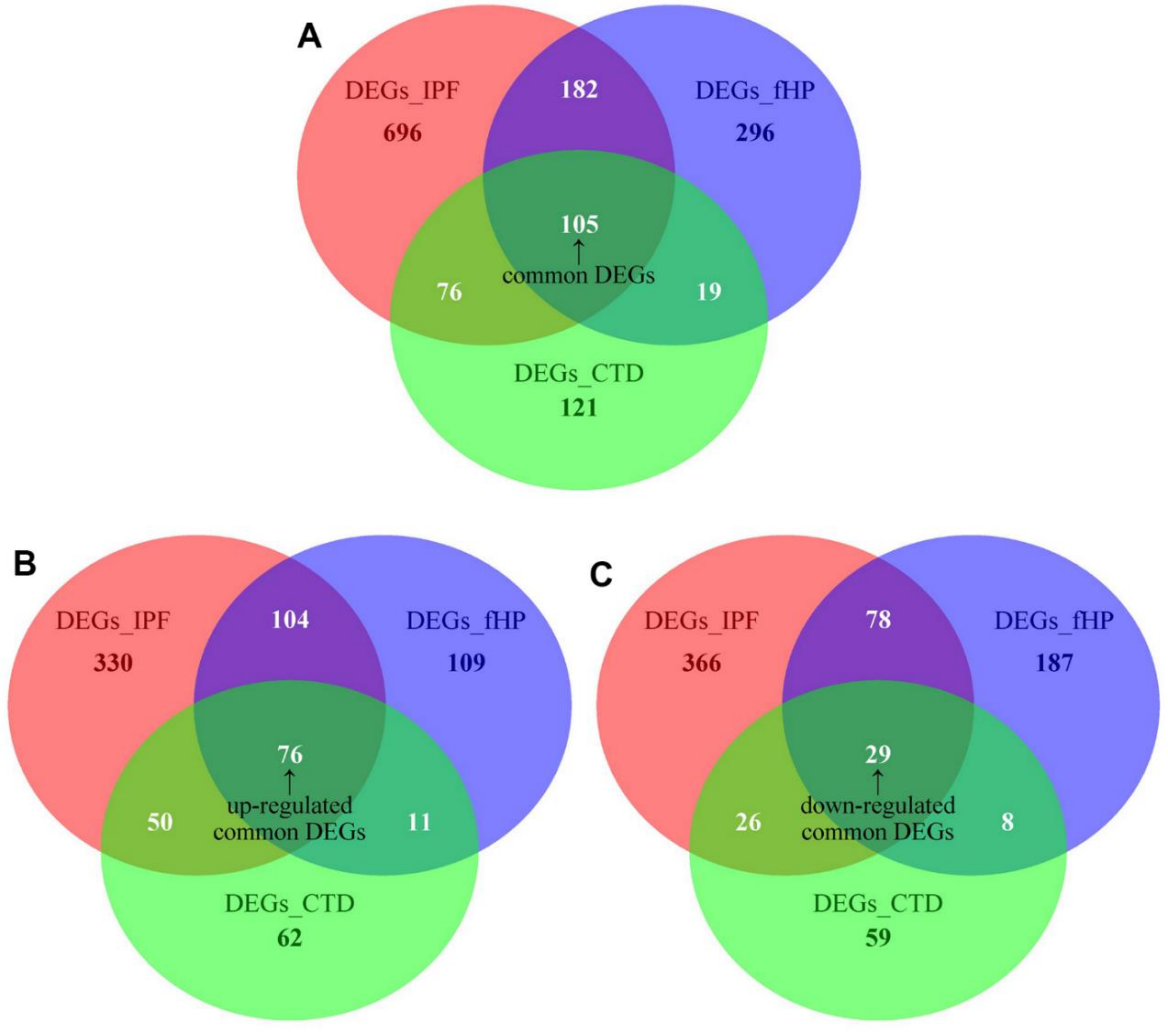
(**A**) Venn diagram demonstrates the common DEGs of fHP, IPF and CTD-ILD. (**B**, **C**) Venn diagrams demonstrate the up-regulated and down-regulated common DEGs of fHP, IPF and CTD-ILD, respectively.

Four independent studies met our inclusion criteria (Table 1) were identified. First, a dataset consisting of 26 patients with CTD-ILD and 13 matched healthy controls was generated by merging two IS datasets: GSE48149 and GSE199152 (Table 2). Concurrently, the fundamental clinical characteristics of the CHP and IPF datasets are presented in Tables 3, 4, respectively. We controlled batch effects and normalized different subsets to ensure data consistency. The results showed that data pre-processing was effective and reliable (Supplementary Figure 1). The results of PCA analysis showed that data pre-processing was effective and reliable (Supplementary Figure 2). In total, 1059 DEGs for IPF, 602 DEGs for fHP, and 321 DEGs for CTD-ILD were identified between patients and healthy controls (Figure 1). We found 105 common DEGs between IPF, fHP, and CTD-ILD, among which 74 DEGs were upregulated, and 29 DEGs were downregulated (Figure 2).

**Table 2:** Clinical characteristics of the merged CTD-ILD data set.

	**CTD-ILD (N=26)**	**Controls(N=13)**	**P-value**
Gender (Male/Female)	9/17	5/8	1
Age at study visit (mean, years)	49.5 ± 10.2	53.3 ± 6.1	0.7510
Smoking history	10/16	4/9	0.7334

**Table 3:** Clinical characters of the fHP data set.

**Characteristic**	**fHP (n = 82)**	**Control (n = 103)**	**P-Value**
Age, years	59.4 ± 10.6	59.9 ± 10.2	0.98
Sex	n = 81	n = 103	0.23
Male	38 (47%)	45 (44%)	
Female	43 (53%)	58 (56%)	
Race	n = 75	n = 103	
Non-Hispanic white	59 (79%)	87 (84%)	0.055
Hispanic	10 (13%)	4 (4%)	—
Asian	4 (5%)	3 (3%)	—
Black	1 (1%)	9 (9%)	—
Other	1 (1%)	0 (0%)	—
Smoke	n = 73	n = 96	0.9
Ever	33 (45%)	43 (45%)	
Never	40 (55%)	53 (55%)	
Sampling method			0.51
Surgical lung biopsy	26 (32%)	41 (40%)	
Transplant	56 (68%)	62 (60%)	
MUC5B genotype			<0.001
GG	52 (63%)	80 (78%)	
GT	29 (35%)	21 (20%)	
TT	1 (2%)	2 (2%)	
Minor allele frequency	0.19	0.12	—

Data are presented as mean ± standard deviation or number (%).

fHP, fibrotic hypersensitivity pneumonitis; yr, years; M, male; F, female.

**Table 4:** Clinical characters of the IPF data set.

**Characteristic**	**IPF (n = 234)**	**Control (n = 188)**	**P-Value**
Age, years	61.4 ± 7.5	55.3 ± 16.8	9.92 x 10^-6^
Sex	n = 234	n = 188	1.11x10^-5^
Male	184 (79%)	110 (59%)	
Female	50 (21%)	78 (41%)	
Race	n = 234	n = 188	0.42
White	200 (85%)	155 (82%)	—
Non-White	34 (15%)	33 (18%)	—
Smoke	n = 234	n = 188	5.7 x10^-3^
Ever	140 (60%)	86 (46%)	
Never	75 (32%)	89 (47%)	
Unknown	19 (8%)	13 (7%)	

Data are presented as mean ± standard deviation or number (%).

IPF, idiopathic pulmonary fibrosis.

### Shared biological pathways in IPF, fHP, and CTD-ILD

**Figure 3:**
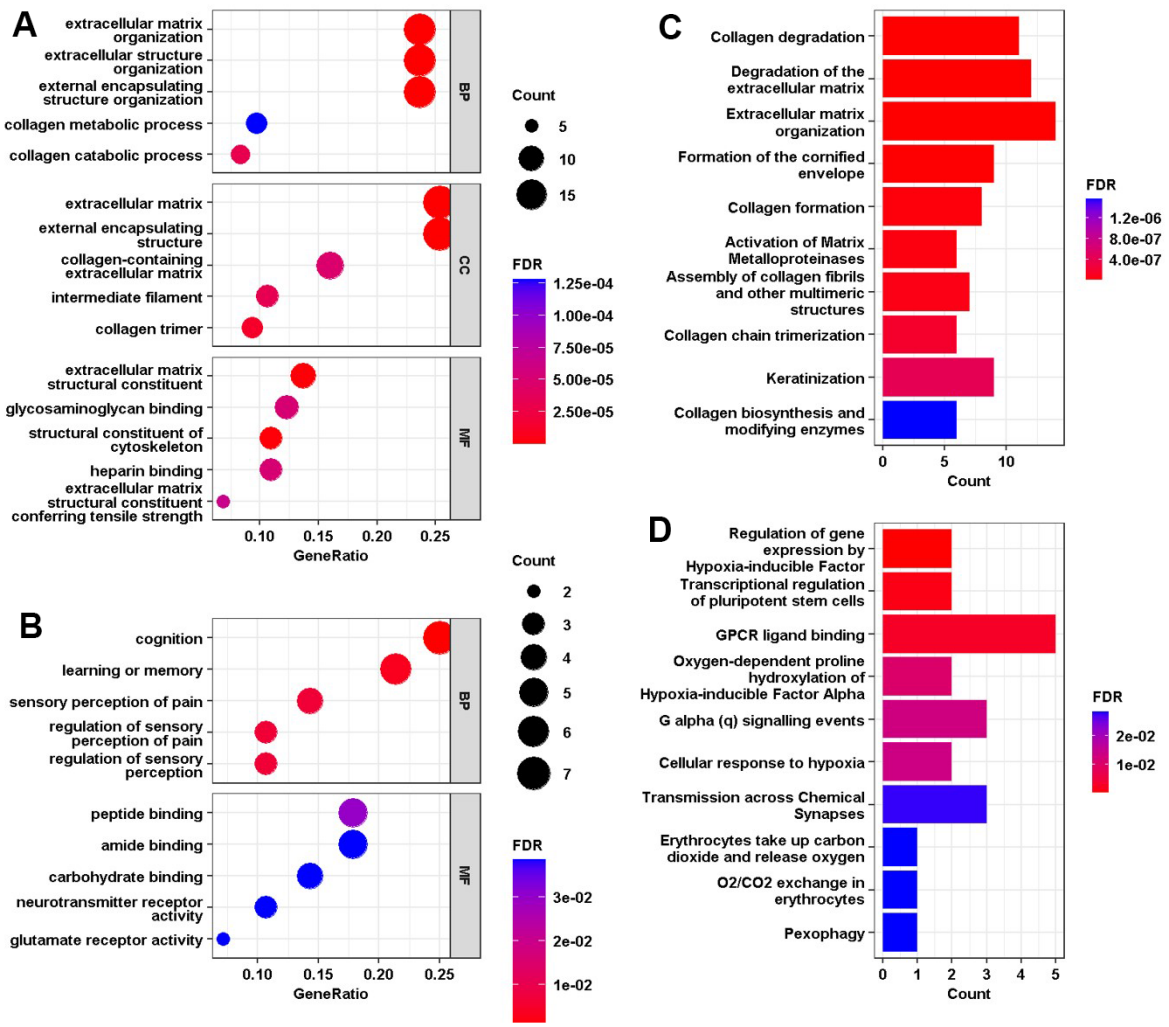
(**A**, **B**) GO enrichment analysis of the up-regulated and down-regulated common DEGs, where the horizontal axis represents the proportion of DEGs under the GO term. Top 5 pathways with most significant P-value were shown and ordered by gene ratio. BP, biological process; CC, cellular component; MF, molecular function. (**C**, **D**) Gene set enrichment analysis of the up-regulated and down-regulated common DEGs, where the horizontal axis represents the number of DEGs under the gene set enrichment analysis (GSEA) terms.

For biological processes, 30 pathways reached statistical significance (Figure 3). Upregulated genes were associated with extracellular matrix changes while downregulated genes were relevant to cognition and sensory perception. For cellular components, upregulated genes were involved in the extracellular matrix, collagen trimming, and intermediate filament. The molecular function significantly associated with common upregulated DEGs was extracellular matrix structural constituent, structural constituent of the cytoskeleton, glycosaminoglycan binding, and heparin binding. Peptide, amide, carbohydrate binding, neurotransmitter receptor activity, and glutamate receptor activity were associated with the enrichment of downregulated genes. More information for GO and DO enrichment analyses is presented in Figure 3A, 3B and Supplementary Figures 3, 4.

Furthermore, the GSEA results demonstrated that the enriched molecular pathways related to the upregulated DEGs were degradation of the extracellular matrix and collagen, formation of the cornified envelope and collagen, activation of matrix metalloproteinases, and keratinization (Figure 3C). Meanwhile, downregulated DEGs were associated with monitoring of oxygen-dependent proline hydroxylation of hypoxia-inducible factor α (HIF-1α), pluripotent stem cells, GPCR ligand binding, Gα (q) signaling, transmission across chemical synapses, O2/CO2 exchange, and pexophagy. These results, particularly the results upregulated DEGs, were consistent with those from GO enrichment analysis, indicating extracellular environment involvement.

### Unique biological pathways in IPF, fHP, and CTD-ILD

**Figure 4:**
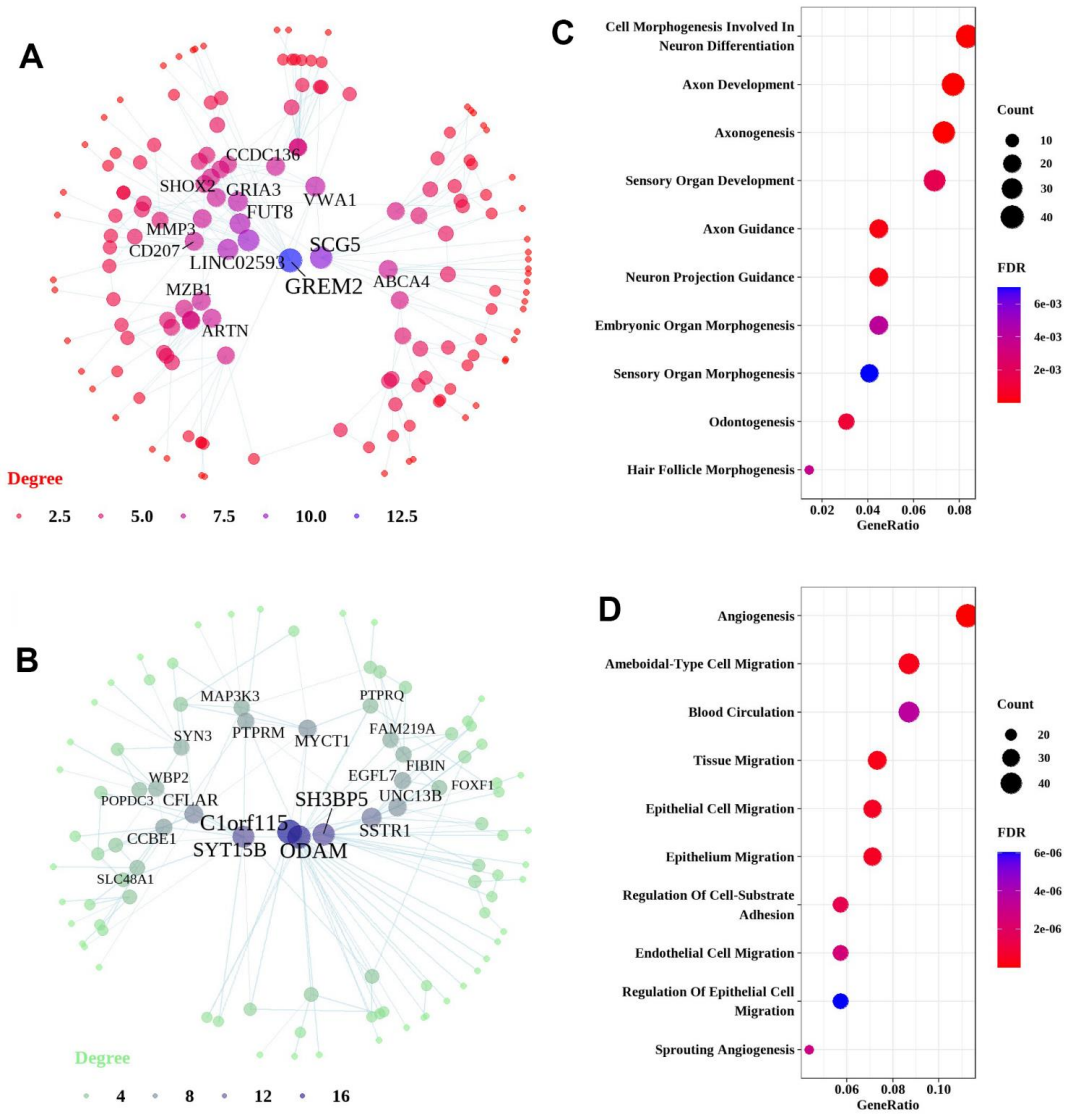
**Network and pathway analyses of DEGs in the lung tissues of IPF patients.** Network and pathways were performed on the dataset from IPF patients compared to healthy controls to identify unique dysregulated pathways in the lung tissues of IPF. (**A**, **B**) Network analysis of up-regulated genes and down-regulated genes. Most significant hub genes, according to degree and betweenness centrality, with the highest number of connections, are enclosed in ovals. (**C**, **D**) Function enrichment analysis of the up-regulated and down-regulated common DEGs, where the horizontal axis represents the proportion of DEGs under the functional terms. Top 5 pathways with most significant P-value were shown and ordered by gene ratio.

Network analysis of upregulated genes identified in the differential analysis of the IPF dataset resulted in a unique network centered on Gremlin 2 (GREM2). GREM2 encodes an antagonist of bone morphogenetic protein (Figure 4A). This network was predominantly enriched in genes associated with neuron-related pathways, including axon development, axonogenesis, axon guidance, and neuron projection guidance (Figure 4C). Network analysis of downregulated genes identified in the differential analysis of the IPF dataset resulted in a particular network centered on chromosome 1 open reading frame 115 (C1orf115) and odontogenic ameloblast-associated protein (ODAM) (Figure 4B). Downregulated genes were predominantly enriched in angiogenesis, epithelial cell migration, and endothelial cell migration (Figure 4D).

**Figure 5:**
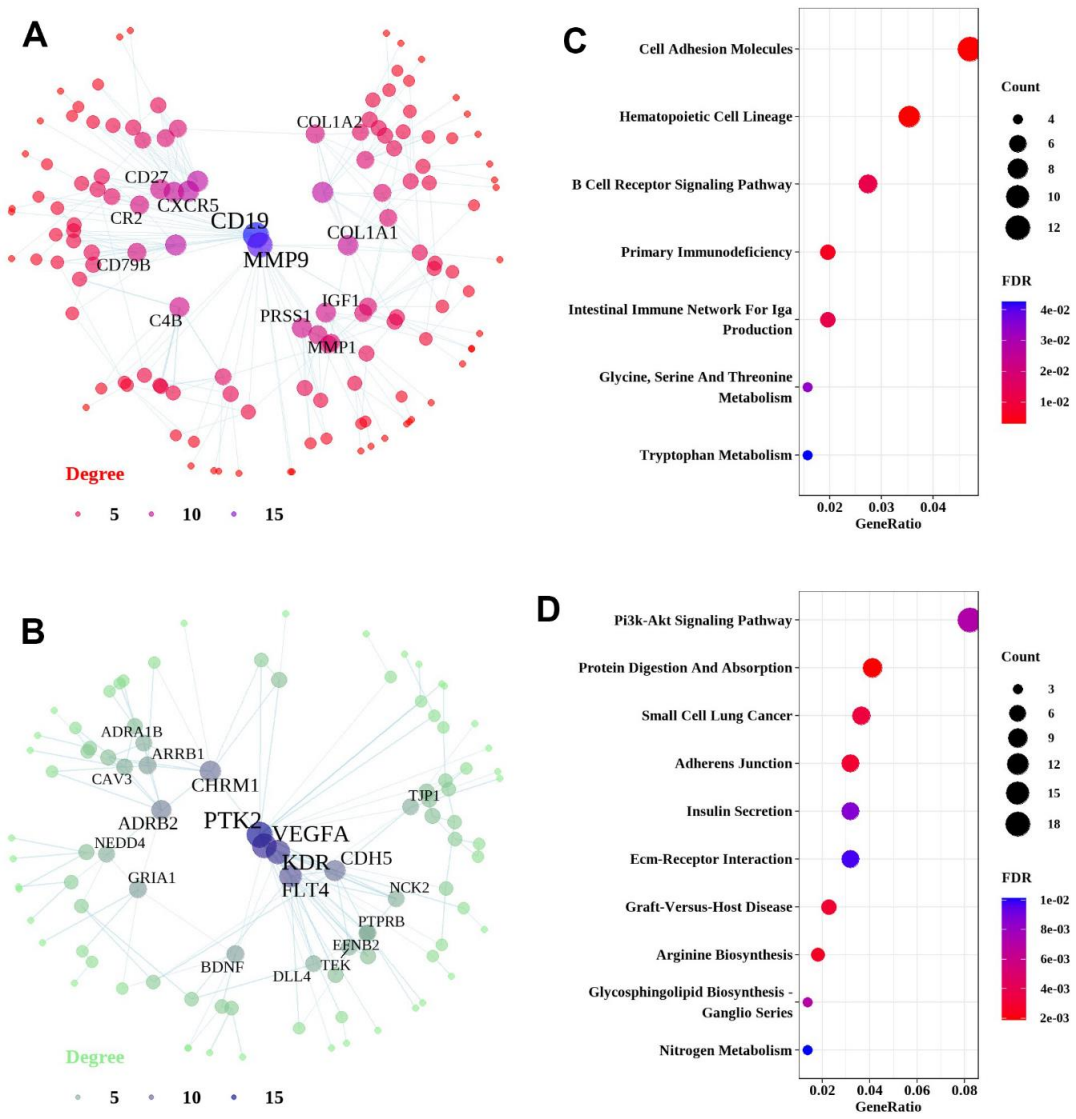
**Network and pathway analyses of common DEGs in the lung tissues of fHP patients.** Network and pathways were performed on the dataset from fHP patients compared to non-demented controls to identify unique dysregulated pathways in the lung tissues of fHP. (**A**, **B**) Network analysis of up-regulated genes and down-regulated genes. Most significant hub genes, according to degree and betweenness centrality, with the highest number of connections, are enclosed in ovals. (**C**, **D**) Function enrichment analysis of the up-regulated and down-regulated common DEGs, where the horizontal axis represents the proportion of DEGs under the functional terms. Top 5 pathways with most significant P-value were shown and ordered by gene ratio.

The same network analysis was performed for the fHP dataset. The unique network of upregulated genes in fHP was centered on the cluster of differentiation 19 (CD19) and matrix metallopeptidase 9 (MMP9) and was enriched predominantly in immune-related pathways (Figure 5A, 5C). Among them, B cell receptor signaling pathway, primary immunodeficiency, and intestinal immune network for IgA production and cell adhesion molecules were the upregulated pathways. Downregulated genes formed a network centered on vascular endothelial growth factor A (VEGFA), protein tyrosine kinase 2 (PTK2), and kinase insert domain receptor (KDR), which were enriched in protein digestion and absorption, arginine biosynthesis, adherens junction, and PI3K/Akt signaling pathway (Figure 5B, 5D).

**Figure 6:**
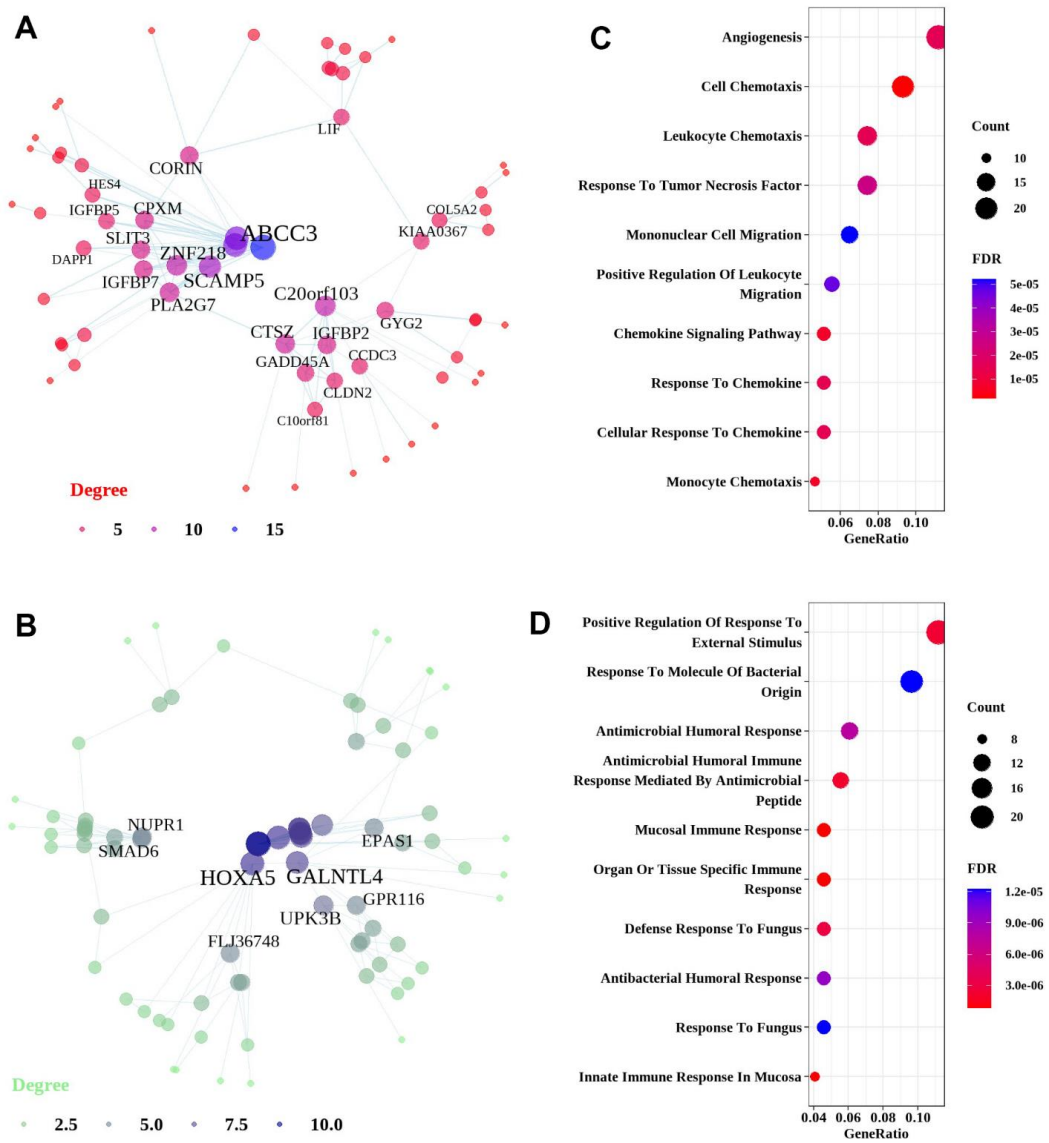
**Network and pathway analyses of common DEGs in the lung tissues of CTD-ILD patients.** Network and pathways were performed on the dataset from CTD-ILD patients compared to non-demented controls to identify unique dysregulated pathways in the lung tissues of CTD-ILD. (**A**, **B**) Network analysis of up-regulated genes and down-regulated genes. Most significant hub genes, according to degree and betweenness centrality, with the highest number of connections, are enclosed in ovals. (**C**, **D**) Function enrichment analysis of the up-regulated and down-regulated common DEGs, where the horizontal axis represents the proportion of DEGs under the functional terms. Top 5 pathways with most significant P-value were shown and ordered by gene ratio.

Analysis of upregulated genes in CTD-ILD resulted in a unique network centered on ATP binding cassette subfamily C member 3 (ABCC3) and was enriched in pathways related to cell chemotaxis (Figure 6A, 6C). Network analysis of downregulated genes in CTD-ILD exhibited a network centered on agouti-related neuropeptide (AGRP) and midline 1 interacting protein 1 (MID1IP1), and enriched predominantly in mucosal immune response (Figure 6B, 6D).

### Identification of regulatory transcription factors

**Figure 7:**
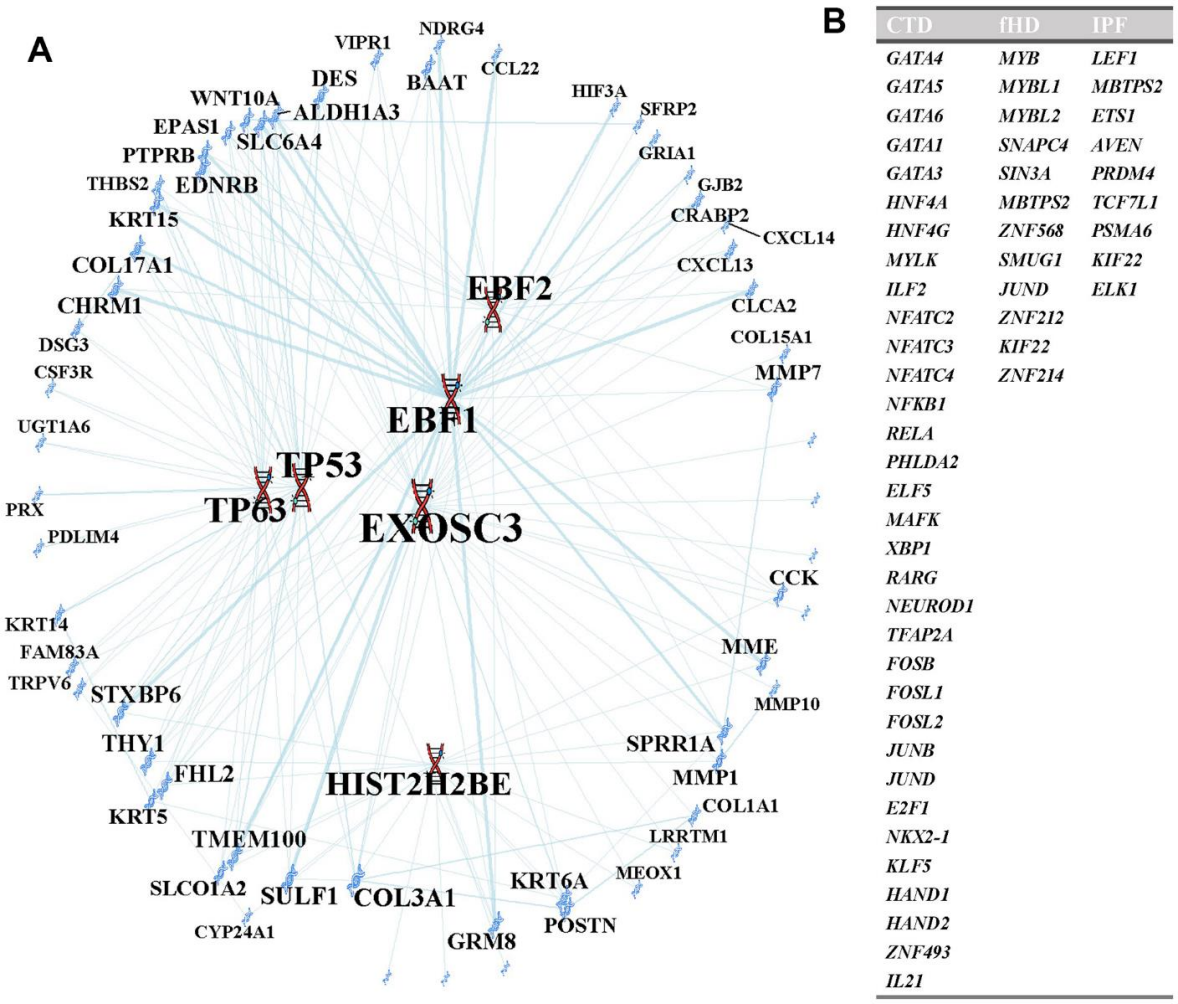
**Transcription factors analysis.** (**A**) TFs regulatory network. TFs were marked in red, and the DEGs were marked in blue. Meanwhile, TFs were enclosed in ovals. (**B**) The transcription factors in this table are unique to each pulmonary fibrosis analysis.

We found six possible critical TFs regulating the expression of these common DEGs including two TFs from the p53 family (Figure 7A). We next investigated unique TFs among the three types of ILDs (Figure 7B). Venn diagram analysis showed that CTD-ILD has the most significant number of specific TFs (33 specific TFs), while fHP and IPF have 12 and 9 specific TFs, respectively.

### Evaluation of tissue-infiltrating immune cells

**Figure 8:**
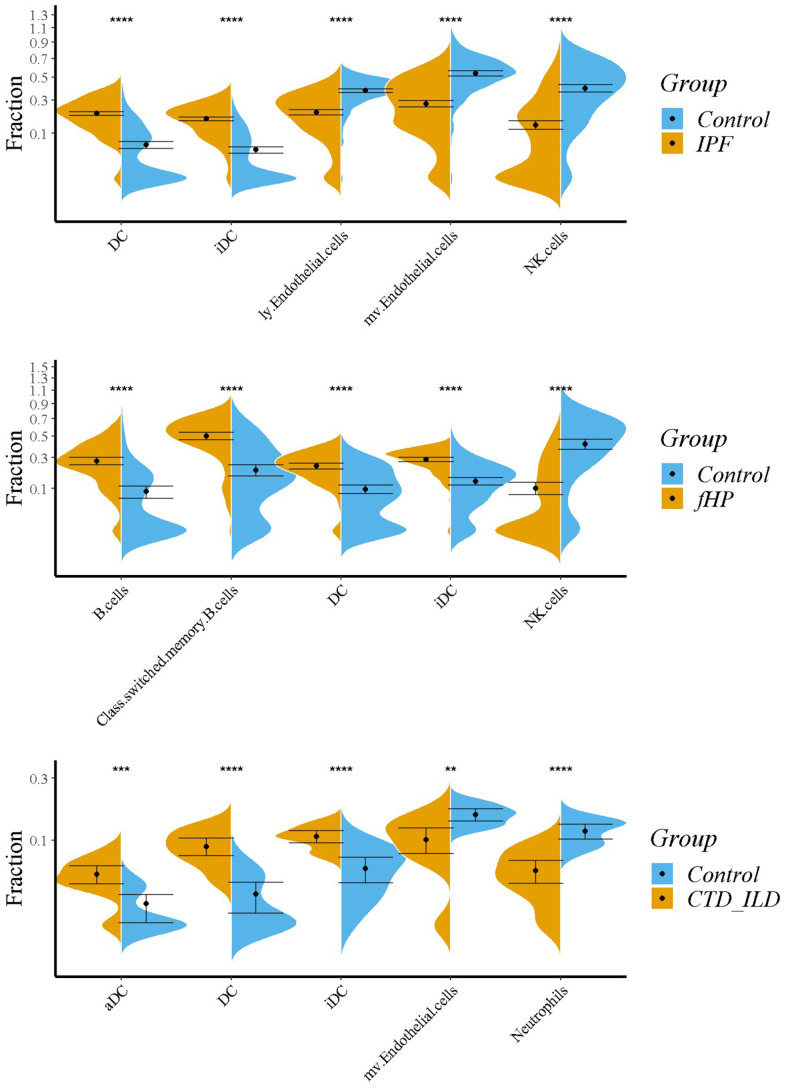
**Immune infiltration analysis by xCell.** Violin plots showing the comparison between 3 ILDs and healthy controls. Top 5 significant variations in immune cell proportions were shown. ns: P ≥ 0.05, *:P < 0.05; **:P < 0.01; ***:P < 0.001; ****:P < 0.0001.

The results of the violin plot showed the top 5 significant differences in immune cell proportions between healthy individuals and patients with IPF, fHP, and CTD-ILD (Figure 8). The increased abundance of DCs, including induced-DCs, was common in the three ILDs. Meanwhile, patients with IPF and fHP exhibited a decrease in natural killer T cell abundance, and patients with IPF and CTD-ILD had decreased abundance of microvascular endothelial cells. Lymphatic endothelial cells were more abundant in IPF; total B cells and class-switched memory B cells were more abundant in fHP; and activated dendritic cells (aDC) and neutrophils were more abundant in CTD-ILD. Furthermore, we observed unique variants of immune cells in the three ILDs (Supplementary Figure 5).

## DISCUSSION

This study revealed that IPF, fHP, and CTD-ILD exhibit comparable downstream molecular patterns but divergent upstream drivers at the transcriptome level. Surprisingly, IPF demonstrated upregulated biological processes associated with neuronal and developmental processes, while fHP showed a prominent involvement of the B cell signaling pathway and intestinal IgA production. CTD-ILD had positive regulation in chemotaxis and weakened response to various pathogens and external stimuli. The diversity of cellular changes may contribute to the different clinical phenotypes, with endothelial cells being more prominent in IPF, total B cells and class-switched memory B cells in fHP, and aDCs and neutrophils in CTD-ILD.

The analysis of shared DEGs revealed that the same molecular mechanisms in IPF, fHP, and CTD-ILD lead to progressive fibrosis. The results of GO enrichment and GSEA further supported this observation, indicating significant alterations in profibrotic ECM changes, such as ECM structure organization and collagen metabolism. Progressive fibrosis in pathological assessment of IPF, fHP, and CTD-ILD can verify these findings [32]. Oxygen-dependent proline hydroxylation of HIF-1α in the GSEA indicated disinhibition of HIF-1α in all three ILDs [33]. Erythrocyte-related processes, including carbon dioxide transfer, oxygen release, and O_2_/CO_2_ exchange were suppressed in all three ILDs, verifying the role of hypoxia in all three ILDs. In the TF analysis, common genes were found to be associated with p53 (encoded by *TP53*) and a p53 family member, p63, suggesting that these TFs may play a role in pulmonary fibrosis. The differential analysis further demonstrated the up-regulation of p53 family members in IPF patients, indicating the activation of the p53 family (Supplementary Figure 6). Former evidences reported that these p53 may promote fibrosis by inducing autophagy resistance in alveolar epithelial cells [34, 35]. The involvement of p53 signaling pathway was previously reported in human lung samples of SSc-ILD and IPF, but not fHP and other CTD-ILDs [10, 36, 37], and its activation was proven to promote lung fibrosis resolution in aged mice [38], which is also reflected in our research. Consistently, our study indicated that p53 may contribute to progressive fibrosis in three ILDs and is a therapeutic target. We displayed the pathway view of collagen degradation to illustrate a shared extracellular environment involvement across three interstitial lung diseases, underscoring a common pathological thread (Supplementary Figure 7).

IPF-specific DEGs were significantly enriched in neuron-related processes like axon guidance (Supplementary Figure 8) and neuron differentiation. Neuronal guidance proteins including netrins, semaphorin, ephrins, and neurophilins are increasingly studied at the interface of injury and repair [39]. It is reported that macrophage-derived netrin-1 (NTN1) drives the development of experimentally induced lung fibrosis via their axon-related functions involving adrenergic nerves remodeling, noradrenaline secretion, and α1 adrenoreceptors [40]. NTN1 deficiency or adrenergic denervation attenuated experimentally induced lung fibrosis [40, 41]. Furthermore, it is confirmed that NTN1 protein expression increased in IPF macrophages and IPF lungs are enriched for noradrenalin [40]. Use of α1 adrenoreceptor antagonists is associated with improved survival in IPF patients [40]. Therefore, our study highlighted that neuron-related processes may play a unique important role in IPF pathogenesis and worth more investigation.

In the analysis of fHP-specific DEGs, three different methods showed B cell receptor signaling pathway (Supplementary Figure 9) as the main culprit. We found that B cells and antibody-related pathways are upregulated and may play more important roles in fHP, though a higher CD4^+^:CD8^+^ ratio and Th1 to Th2 switch are considered to be important in the pathogenesis of fHP in former studies [42]. Consistently, prior study has reported the predominance of B cells in the transcriptome of most severely affected lung zones [43]. CD19, CXCR5, and CCR7 were identified as key regulators of fHP, all of which are all associated with B cell response. Furthermore, the active TFs in HP, such as Myb proto-oncogene protein (MYB), have been implicated in B cell differentiation under specific circumstances [44]. Specifically, increased IgA production and B cell class switching were dominant in fHP, while increased IgA levels were only reported in bronchoalveolar lavage of HP patients before [45, 46]. IgA can possibly induce lung injury by forming immune complexes [47]. Our results illuminated the role of B cells in fHP. This may also help the diagnosis of fHP, as fHP patients are frequently misdiagnosed as IPF or CTD-ILD due to their similar radiologic and/or histopathologic findings.

We also demonstrated that activated DCs play an important role in the pathogenesis of pulmonary fibrosis associated with CTD-ILD. Enrichment analysis showed that chemokine-related pathways (Supplementary Figure 10), particularly mononuclear cell-related processes are active in CTD-ILD. DEGs including CCL19, CCL22, CCL2, and CCL18 were related to DC’s function. Though total DC abundance was elevated in all three ILDs, activated DCs infiltrated the CTD-ILD lung tissue and were a possible source or target of these chemokines [48]. Previous studies primarily attributed CTD-ILD to autoantibodies and resultant dysregulation of innate immunity. However, the specific role of aDCs remains largely unexplored [49, 50]. In contrast to fHP, in CTD-ILD, DCs are activated by endogenous antigens originating from collagen fibers, neutrophil extracellular trap, or immune complexes [51]. Notably, targeting activated follicular DC and inducing DCs’ tolerance has shown promise in alleviating autoimmune conditions [52, 53]. Consequently, interventions aimed at modulating aDCs or chemotaxis in the context of CTD-ILD may hold therapeutic potential for improving patient outcomes.

The strengths of this study were inclusion of most common subtypes of fibrotic ILD and integrated analysis. IPF, fHP, and CTD-ILD have similar pulmonary fibrosis but different causes, allowing for exploration of diverse fibrosis pathways. Additionally, the study analyzed data on different levels, including signaling pathways, protein interactions, immune infiltration, and TFs.

However, there were some limitations to this study. First, the number of samples was not equal among groups because lung tissue from CTD-ILD patients was relatively rare. Second, some subtypes of CTD-ILD, like idiopathic inflammatory myopathy associated-ILD and primary Sjögren syndrome-associated ILD, were missing, though two most prevalent subtypes, SSc- and RA-ILD were included.

In summary, the objective of this study was to compare distinct molecular patterns among three common types of fibrotic ILDs. Transcriptome data from public databases was collected and integrated. 1059 DEGs for IPF, 602 DEGs for fHP, and 321 DEGs for CTD-ILD were identified, and 105 DEGs were found to share among these 3 ILDs. PPI network and functional enrichment were conducted to identify functional modules within each set of DEGs. Interestingly, a shared ECM-related module among 3 ILDs was discovered and possibly regulated by p53 family members. Neuronal response seems to be involved in IPF and B cell signaling pathway was found active in fHP, based on functional enrichment of disease-specific DEGs. Computational immune filtration was performed to predict the cellular changes in the lung tissue. The results indicated an enrichment of aDCs in CTD-ILD, while B cells seemed to dominate in fHP, which was consistent with results from functional enrichment. These molecular and cellular discrepancies may underpin the observed clinical diversity among ILDs. It is imperative to note that the pathways and biomarkers identified herein warrant further validation through experimental research and clinical trials to confirm their relevance and applicability.

## Supplementary Material

Supplementary Figures

Supplementary Table 1

Supplementary Table 2

Supplementary Table 3

Supplementary Table 4

Supplementary Tables 5 and 6

Supplementary Table 7

Supplementary Table 8

Supplementary Table 9

Supplementary Table 10
