# Supplementary material for: Transcriptomic and network analysis identifies shared and unique pathways and immune changes across fibrotic interstitial lung diseases
Source: Aging (Albany NY). 2024 Feb 12;16(4):3200–30. doi: 10.18632/aging.205530 (PMC10929820; doi:10.18632/aging.205530)
Supplement: Supplementary Figures [file aging-16-205530-s001.pdf]

SUPPLEMENTARY FIGURES

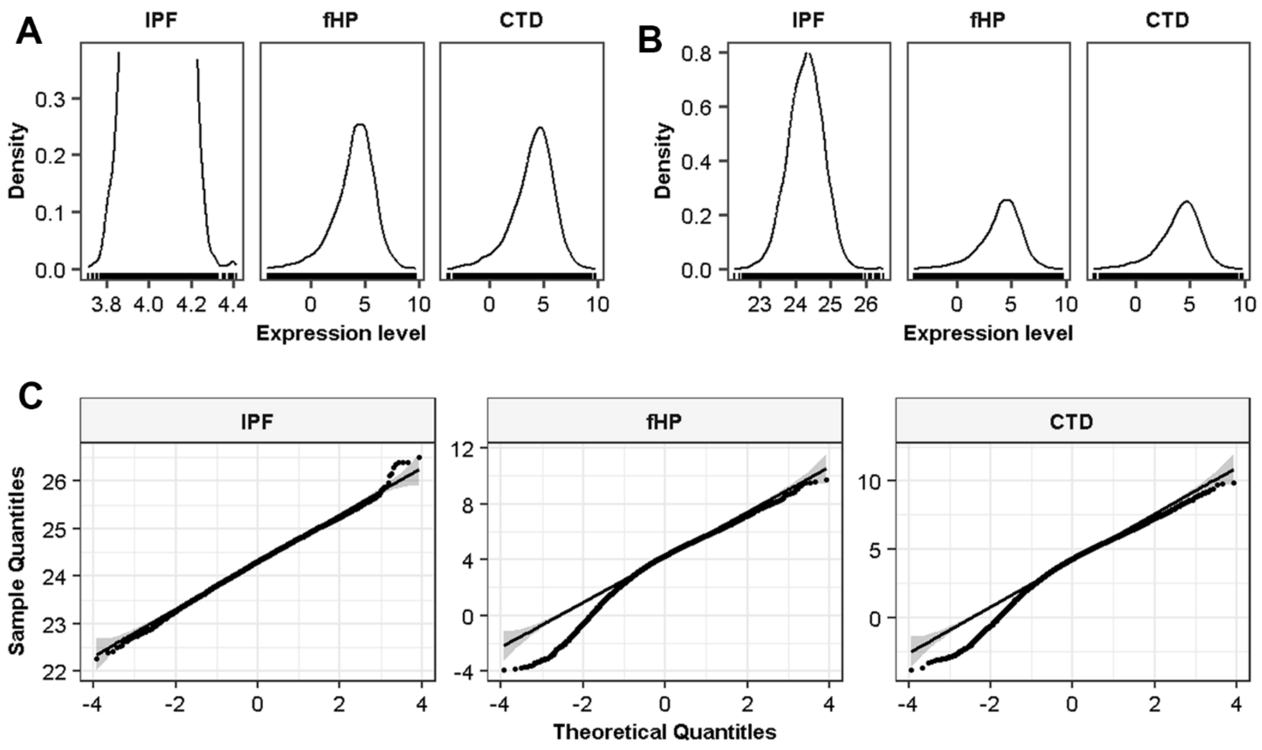

Supplementary Figure 1. The correction of datasets used in this study. (**A**, **B**) The density plots of IPF, fHP and CTD-ILD datasets before (**A**) and after (**B**) normalization and removing batch-effects. (**C**) The Q-Q plot of the three datasets after correction.

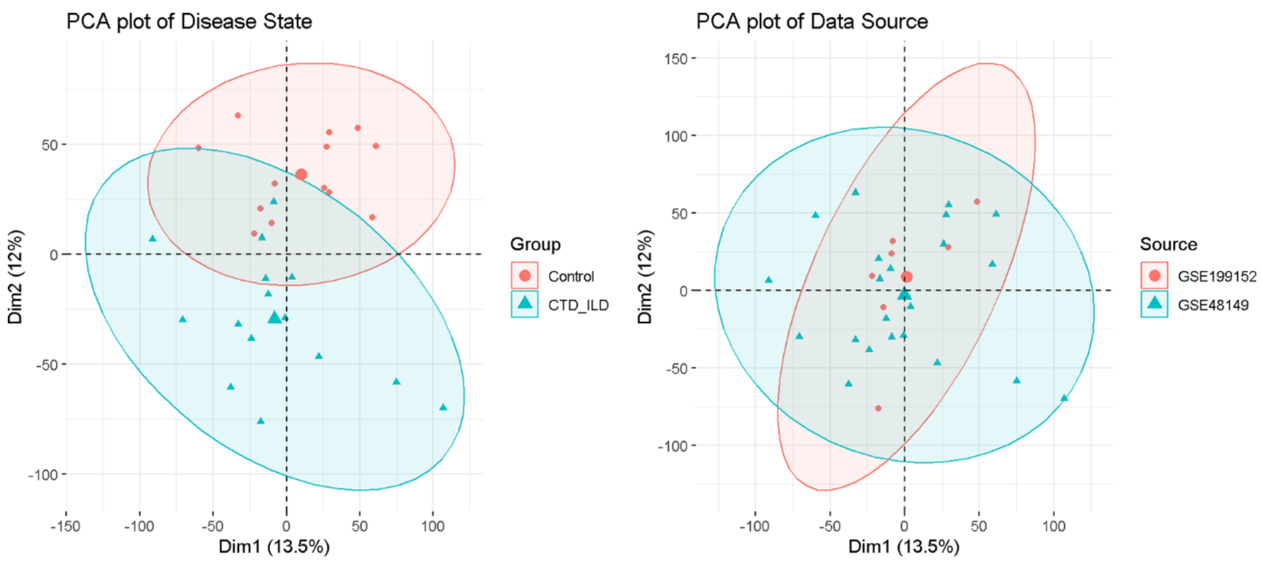

Supplementary Figure 2. The principal component analysis (PCA) plot of the merged dataset by disease state and the original datasets.

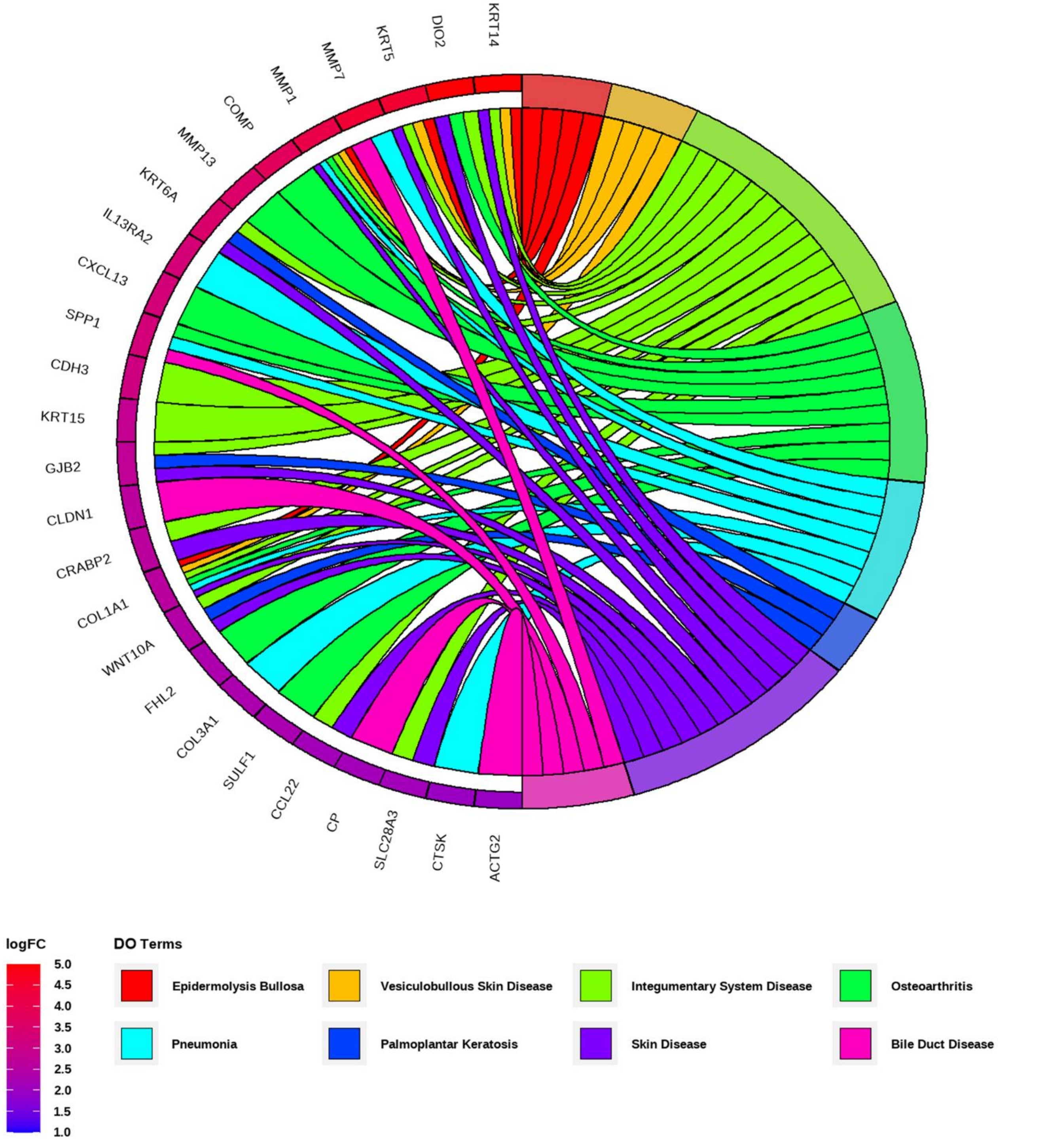

Supplementary Figure 3. DO enrichment analysis. Chord diagram showed the correlation between diseases and common down-regulated DEGs, with different colors corresponding to different DO terms.

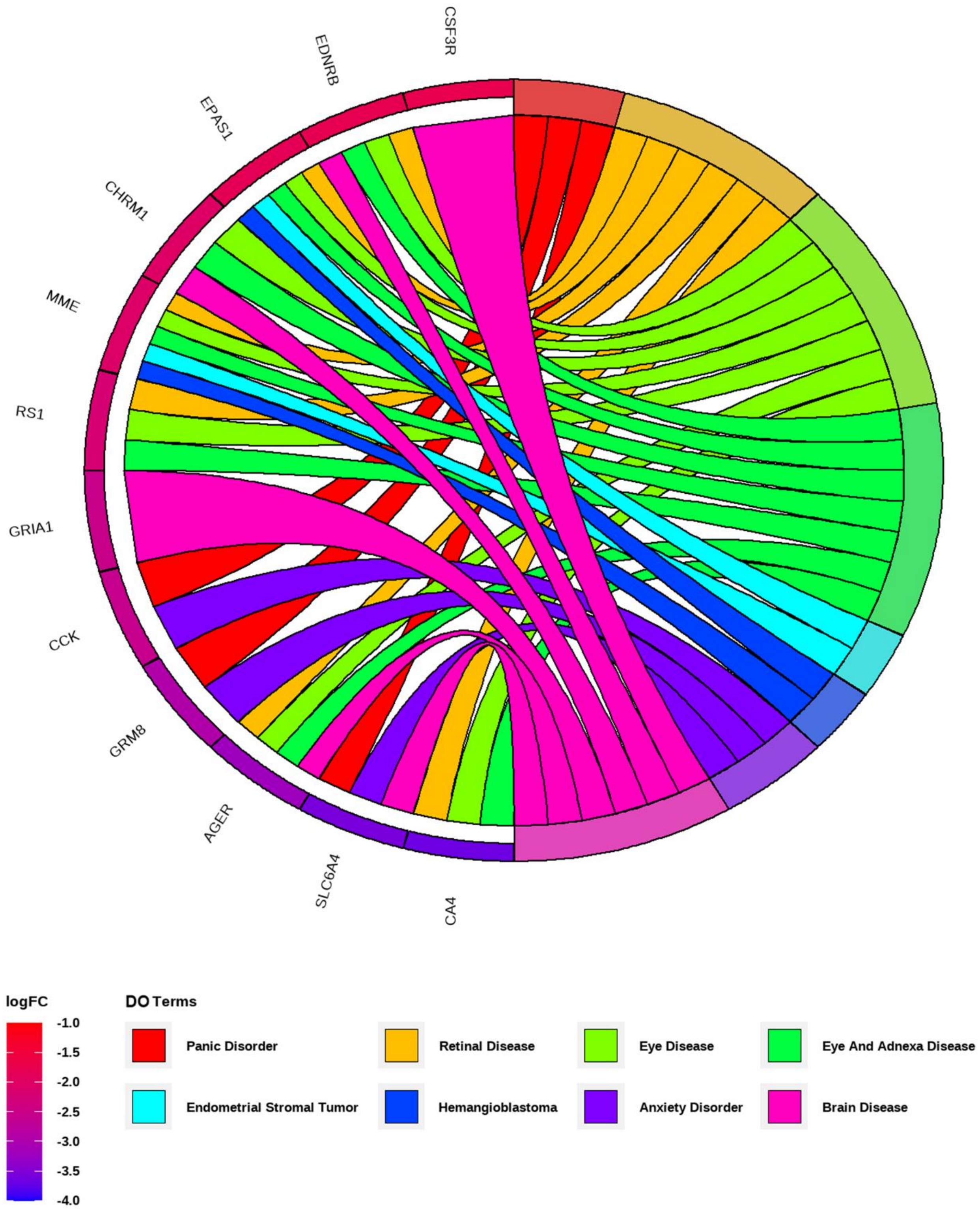

Supplementary Figure 4. Immune infiltration analysis. Violin diagram showing three diseases' unique proportion variation of immune cells obtained using the xCell. *:P < 0.05; **:P < 0.01; ***:P < 0.001; ****:P < 0.0001

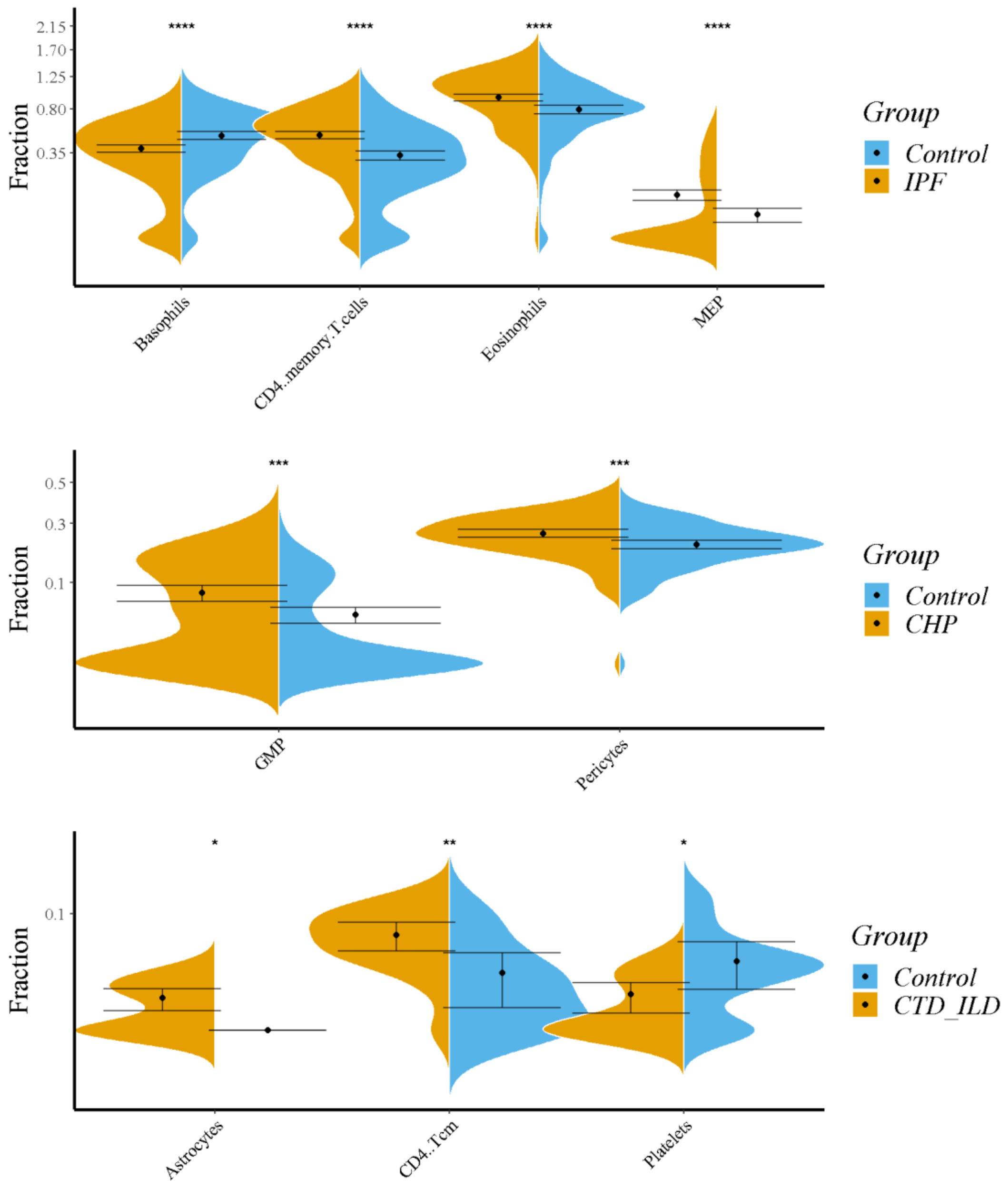

Supplementary Figure 5. Activation of the p53 family in IPF patients. Boxplot showing the up-regulation of p53 family members in patients with idiopathic lung fibrosis. ****:P < 0.0001.

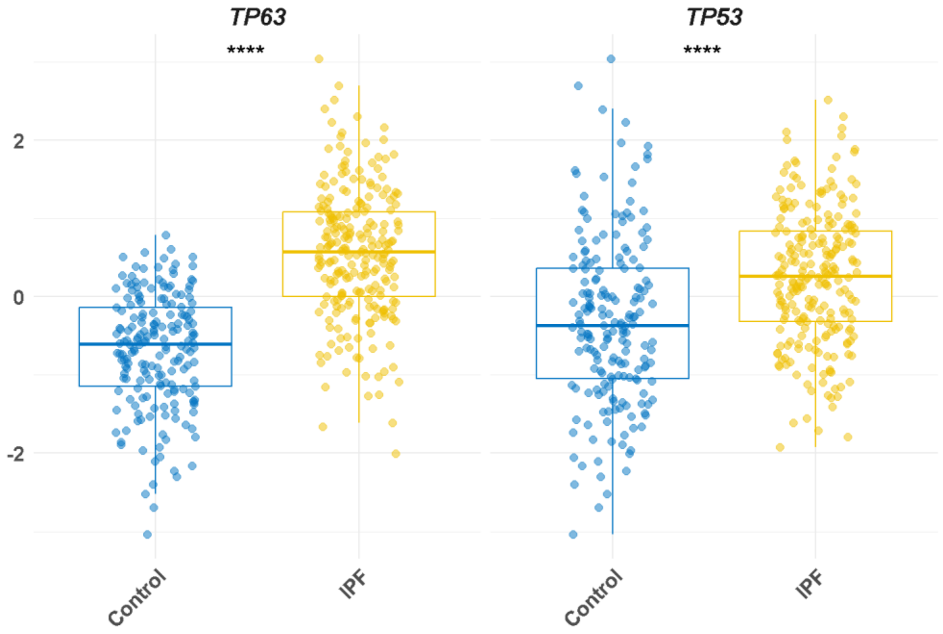

Supplementary Figure 6. Activation of the p53 family in IPF patients. Boxplot showing the up-regulation of p53 family members in patients with idiopathic lung fibrosis. ****:$P < 0.0001$.

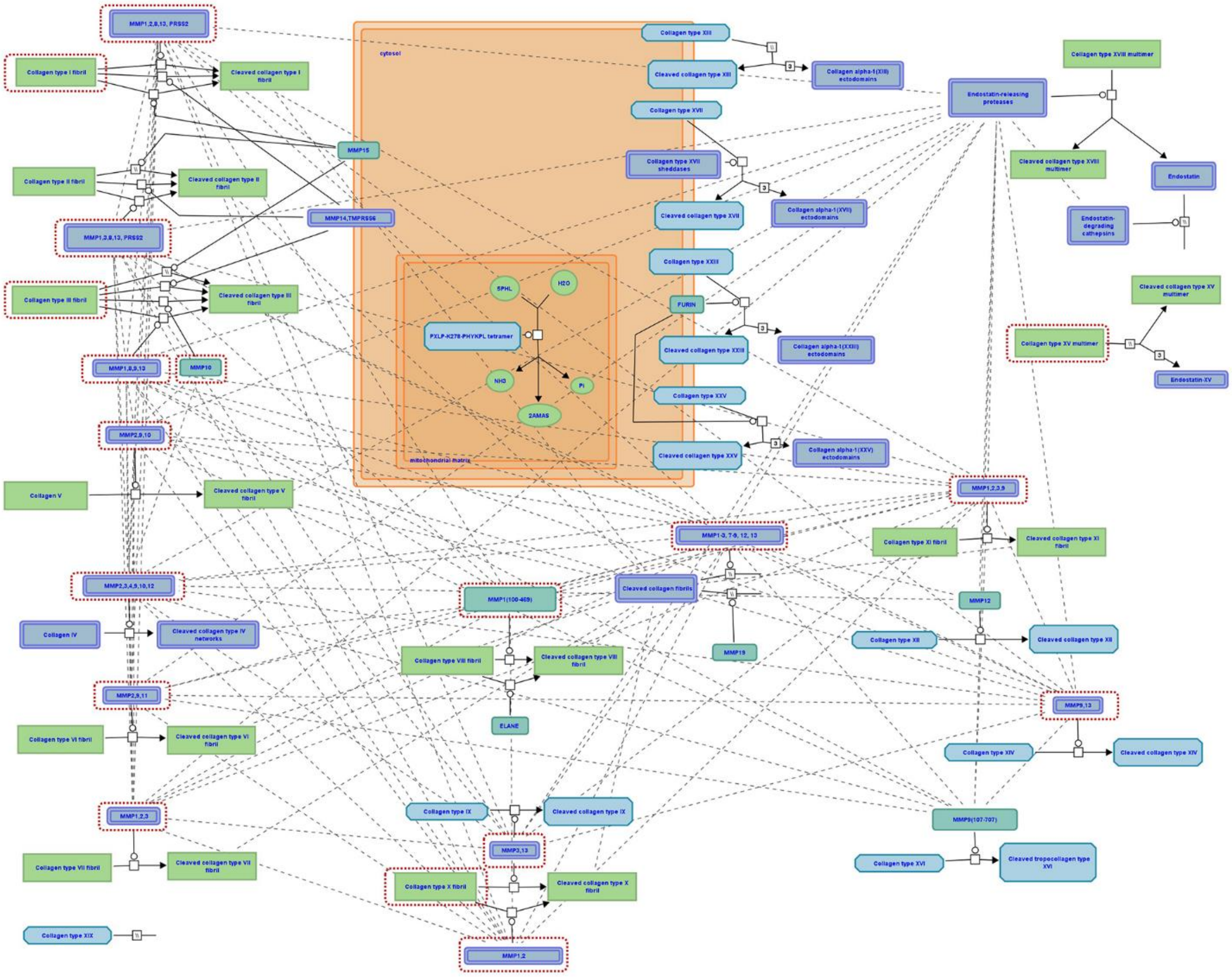

Collagen degradation

Supplementary Figure 7. Pathway view of collagen degradation. The common DEGs were emphasized with red box.

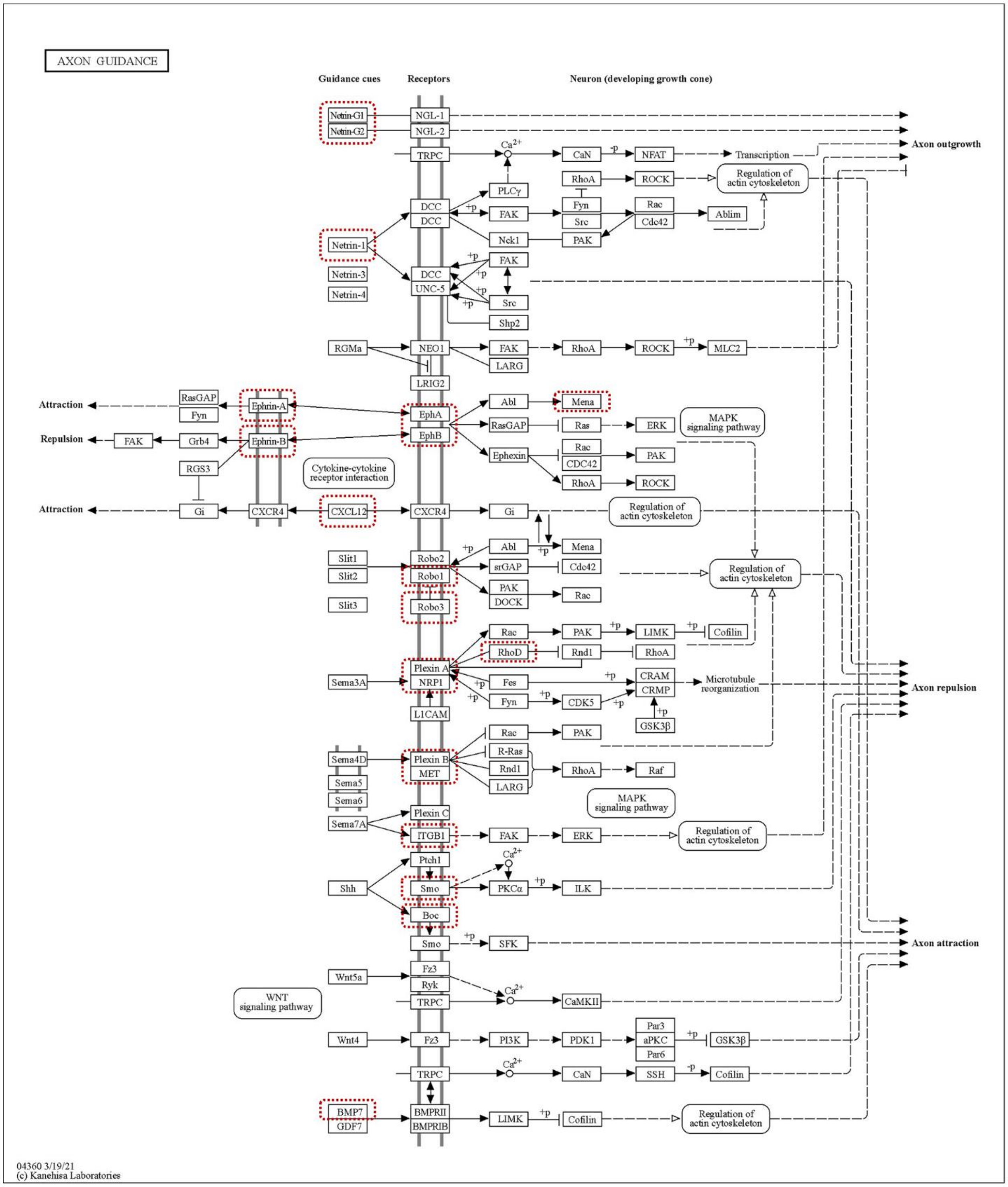

Supplementary Figure 8. Pathway view of axon guidance. The IPF-specific DEGs were emphasized with red box.

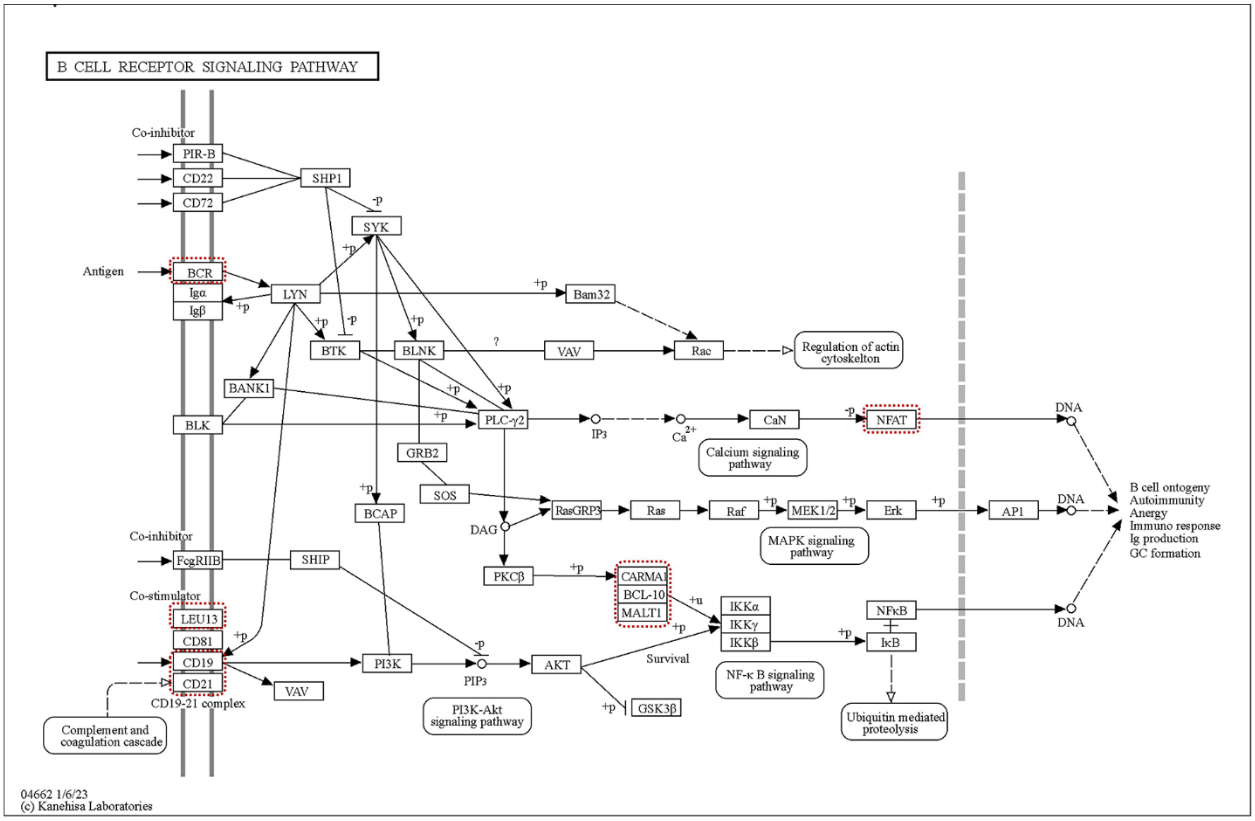

Supplementary Figure 9. Pathway view of B cell receptor signaling. The CHP-specific DEGs were emphasized with red box.

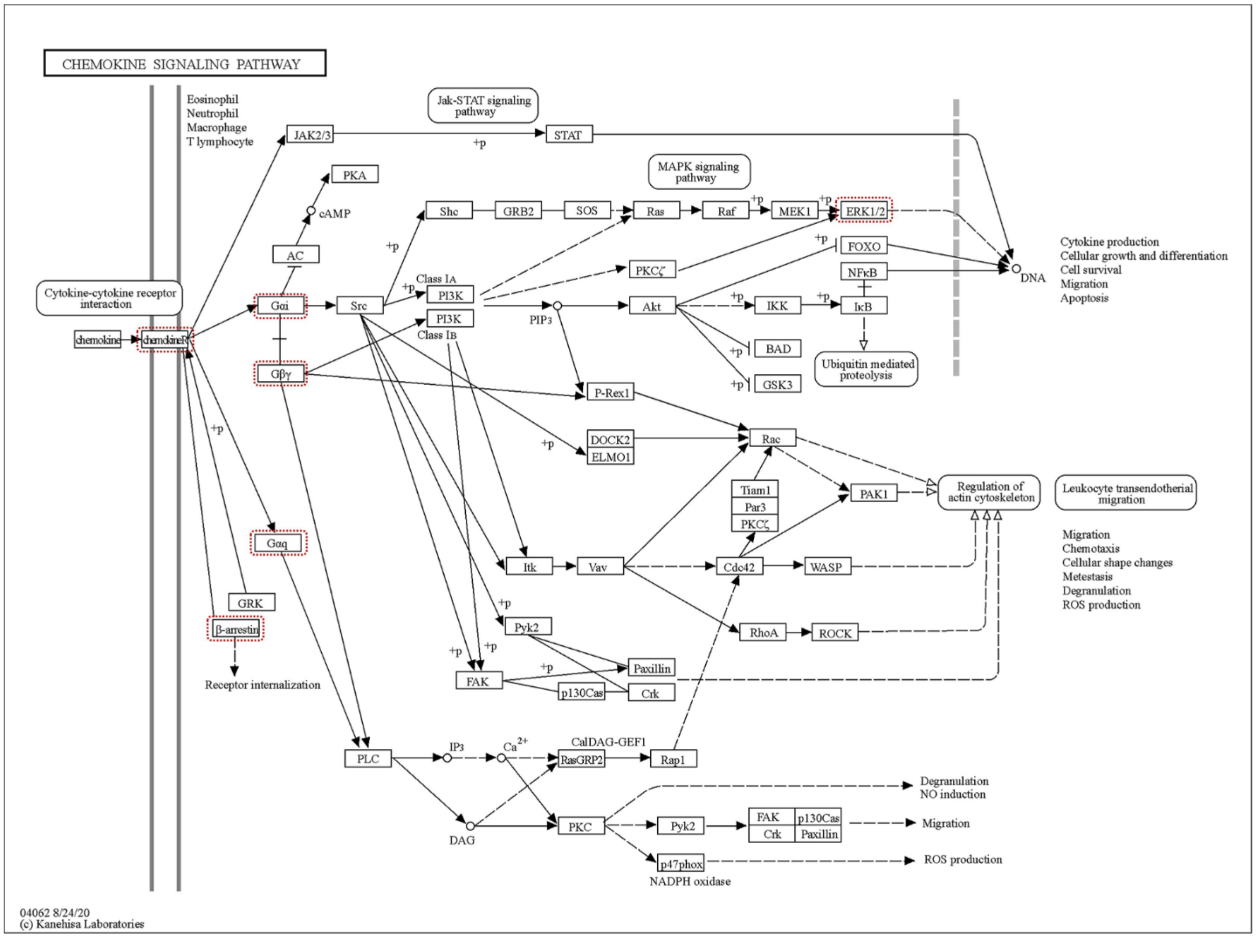

Supplementary Figure 10. Pathway view of chemokine signaling. The CTD_ILD-specific DEGs were emphasized with red box.
