# Supplementary material for: Transcriptomic and network analysis identifies shared and unique pathways and immune changes across fibrotic interstitial lung diseases
Source: Aging (Albany NY). 2024 Feb 12;16(4):3200–30. doi: 10.18632/aging.205530 (PMC10929820; doi:10.18632/aging.205530)
Supplement: Supplementary Tables 5 and 6 [file aging-16-205530-s006.pdf]

Supplementary Table 5. Up-regulated common differentially expressed genes of IPF, fHP and CTD-ILD.

hgnc_symbol	logFC_IPF	logFC_CHP	logFC_CTD.ILD
ACTG2	1.16867332289099	1.06884319181345	1.57264761554731
ALDH1A3	1.37938443626019	1.203935667529	2.02540445237465
BAAT	1.50486346899843	2.11337420730513	2.15145213982184
CCL22	1.21217326638706	1.32230959545926	1.46059285766171
CD1A	1.28563576066025	1.19629725112951	1.30184435915926
CD207	1.28503231545761	1.32747024212248	1.4483832918451
CD24	1.3567283865497	1.30857017633235	1.35159383429003
CD79A	1.1001129187577	1.60528405537751	1.31164920456388
CDH3	1.55254377511596	1.77422180393457	1.77715247941456
CILP2	1.43081105473093	1.63602244229272	1.08019620698807
CLCA2	1.1889021961	1.35272328038441	1.90153630382498
CLDN1	1.35303152912343	1.26599260803912	2.03637389720905
COL10A1	1.41114064628794	2.15572235346214	2.02614774212009
COL15A1	1.32217306566972	1.16719339312545	2.03491115864745
COL17A1	1.56579578040413	3.11378506173711	2.09905827748447
COL1A1	1.19967078320226	1.36940141859273	2.09054631335168
COL22A1	1.42887166919087	1.08783036913002	1.07928914726629
COL3A1	1.16138217513617	1.19792189825317	1.96960198466899
COMP	1.44021388998733	2.18515988472482	2.29253797286178
CP	1.25006262921802	1.59861653042657	1.15130529617468
CR2	1.12132181659738	4.76726574883564	1.20100749441066
CRABP2	1.46726880643017	1.68475783603254	1.40603848092354
CST1	1.14702917580896	4.67484799147212	2.41315816141039
CST2	1.32344449403933	2.43414705816646	2.09366865964664
CTHRC1	1.40382311258675	1.40711633966439	1.98177353779443
CTSK	1.30885109655958	1.20945785400863	1.27868864164933
CXCL13	1.28943496908849	2.5439019027623	1.65581870981868
CXCL14	1.44220942925456	1.81851882996128	1.46659837028222
CXCL6	1.14205998213226	1.42926109616135	1.51310015479465
CYP24A1	1.38183581653317	2.91870164219946	1.45646381062104
DES	1.15760796003788	1.05027649574864	1.18699183415991
DIO2	1.50085088162593	4.84026100872179	1.7024286569945
DSG3	1.38275286113405	1.92086551148894	1.35386259338207
FAM83A	1.32648290606239	1.00390407052197	1.36762517730964
FHL2	1.45987116429258	1.04584658659112	1.80808977685406
FNDC1	1.44169186308349	2.3074685642745	1.39798967421432
GJB2	1.38939930334731	1.90585410149485	1.44066489248103
HS6ST2	1.53810380412916	1.29069179835172	1.04641986611265
IGFL1	1.4173567987978	4.10096599631326	1.30994741132254
IGFL2	1.50505031248118	4.10850318037433	2.20639209105057
IL13RA2	1.50103761252354	1.65577949471032	2.25078732597388
KRT14	1.48071175889854	4.79832833957068	1.95558847504554
KRT15	1.30519460842457	1.82896466456427	1.68717749254765
KRT16	1.31863430545208	4.60334339417622	1.15553978505549
KRT17	1.45526856223259	2.38141883794919	3.08328692257748
KRT5	1.345186402039	2.36519549370348	3.13204675919996
KRT6A	1.27725887623621	2.34136032196097	2.06608901185009

KRT6B	1.17825127028074	2.79711661913147	1.70233098181525
LRRTM1	1.34460832671252	2.86863997930985	1.42821470996562
MDK	1.28116659256428	1.14996875744481	1.49032602470256
MEOX1	1.2194108341199	2.47679192252652	1.317949319174
MMP1	1.41926330419899	2.36944621973944	2.52411096843166
MMP10	1.25697335482946	1.85519717382516	1.92540471014862
MMP11	1.49021125416502	1.87211172610846	1.88099243180746
MMP13	1.48302296385838	3.44614767062312	1.2528582962855
MMP7	1.51474262024814	2.29674819012599	2.82787276324862
PCP4	1.39770370221466	3.49108552294338	1.91730096143447
PDLIM4	1.42251447811563	1.49837642836773	1.23591530117167
POSTN	1.34102947292959	1.42996786093157	1.71850107261227
POU2AF1	1.2747842380671	1.26539652745947	1.21263194662377
SCG5	1.49102062977927	2.95977608871547	1.4585972748726
SERPINB5	1.36125280521898	2.42067874661796	1.9944708717494
SERPIND1	1.24819123091871	1.53196417422222	1.96175863645607
SFRP2	1.38283433251574	1.55744901541122	2.37094794321419
SLC28A3	1.28073117579086	1.02551876584639	1.74312735750169
SLN	1.20043899312754	1.62301222630393	1.09394416395194
SPP1	1.20157691284556	1.49184373727737	2.97845347776939
SPRR1A	1.337886445773	4.26712471227044	1.13264020888067
SULF1	1.43755354979499	1.07367922897018	1.70103084623035
THBS2	1.18709534646714	1.06521212072047	1.68326877480194
THY1	1.3345079226186	1.86940548944793	2.74004298678487
TRPV6	1.35762436742283	1.15224496082757	1.20221419882409
TUBB3	1.36740853052801	2.53274536700379	1.30448508092816
UCN2	1.33588447389693	1.31905940884364	1.13027254748341
UGT1A6	1.32118085405306	2.82876121457458	1.58714934524663
WNT10A	1.37898853272126	1.45352136155049	1.52635758438604

Supplementary Table 6. Down-regulated common differentially expressed genes of IPF, fHP and CTD-ILD.

hgnc_symbol	logFC_IPF	logFC_CHP	logFC_CTD.ILD
AGER	-1.34899997867565	-1.42199951903428	-2.64609928429044
BTNL9	-1.42003716481868	-1.62515713982911	-2.34544821942771
CA4	-1.4501328267098	-1.5884186896536	-2.90308555733666
CCK	-1.120331223529	-2.62561468464483	-1.10951595760675
CHRM1	-1.16493901069145	-1.45895209169062	-1.19574462535598
CRTAC1	-1.22207832130913	-1.1324549012924	-1.66030534025204
CSF3R	-1.23097381642726	-1.15494893358054	-1.0504694882291
EDNRB	-1.30050312761451	-1.1119168035004	-1.07601469183884
EPAS1	-1.28412788703569	-1.26627830723914	-1.02230942444717
FCN3	-1.26412115067964	-1.24178649339451	-2.57438309243446
GALNT13	-1.56703692435974	-1.93826251238563	-1.23444085378394
GRIA1	-1.33586041118294	-1.89998969983665	-1.23841567821961
GRM8	-1.12127198304424	-2.89794990829132	-1.32327719649941
HIF3A	-1.3482116537219	-1.36676416826069	-1.14455075292685
HTR3C	-1.16608921452496	-1.35575762501819	-1.42230123302719
ITLN2	-1.46698772149713	-3.02657947692901	-3.38646612110895
KLRF1	-1.22424721414267	-1.64203657219513	-1.04733916449086
MME	-1.32923029628891	-1.33764545145942	-1.17963698890775
NDRG4	-1.22391023699594	-1.41714821190263	-1.31730113529117
PNMT	-1.1658308453927	-1.0634866396352	-1.53618040595259
PRX	-1.35922208820321	-1.27536241010644	-1.60625736257521
PTPRB	-1.36952057540505	-1.02877211145758	-1.20802965804873
RGS9BP	-1.2561647656702	-1.09809486301492	-1.29897202906918
RS1	-1.33465448139793	-1.05537623233956	-1.72487566322996
SLC6A4	-1.17726120301447	-2.32365869095908	-2.339236094182
SLCO1A2	-1.3625353577266	-3.28361942036096	-1.11892598862302
STXBP6	-1.33747791880127	-1.52101017786342	-1.1840624542589
TMEM100	-1.18507102935744	-1.29489581247938	-2.3646363303669
VIPR1	-1.23025374592456	-1.70707144428038	-2.30090054973427
